# Process Evaluation of a Workplace Integrated Care Intervention for Workers with Rheumatoid Arthritis

**DOI:** 10.1007/s10926-015-9624-z

**Published:** 2016-01-25

**Authors:** M. van Vilsteren, C. R. L. Boot, A. E. Voskuyl, R. Steenbeek, D. van Schaardenburg, J. R. Anema

**Affiliations:** 1 Department of Public and Occupational Health, EMGO Institute for Health and Care Research, VU University Medical Center, Room BS7-C573, Van der Boechorststraat 7, 1081 BT Amsterdam, The Netherlands; 2 Body@Work, Research Center on Physical Activity, Work, and Health, TNO-VU University Medical Center, Amsterdam, The Netherlands; 3 Department of Rheumatology, VU University Medical Center, Amsterdam, The Netherlands; 4 TNO Work, Health and Care, Leiden, The Netherlands; 5 Jan van Breemen Research Institute|Reade, Amsterdam, The Netherlands; 6 Research Center for Insurance Medicine AMC-UMCG-UWV-VU University Medical Center, Amsterdam, The Netherlands

**Keywords:** Rheumatoid arthritis, Intervention studies, Work

## Abstract

*Purpose* To perform a process evaluation of the implementation of a workplace integrated care intervention for workers with rheumatoid arthritis to maintain and improve work productivity. The intervention consisted of integrated care and a participatory workplace intervention with the aim to make adaptations at the workplace. *Methods* The implementation of the workplace integrated care intervention was evaluated with the framework of Linnan and Steckler. We used the concepts recruitment, reach, dose delivered, dose received, fidelity and satisfaction with the intervention. Data collection occurred through patient questionnaires and medical records. *Results* Participants were recruited by sending a letter including a reply card from their own rheumatologist. In total, we invited 1973 patients to participate. We received 1184 reply cards, and of these, 150 patients eventually participated in the study. Integrated care was delivered according to protocol for 46.7 %, while the participatory workplace intervention was delivered for 80.6 %. Dose received was nearly 70 %, which means that participants implemented 70 % of the workplace adaptations proposed during the participatory workplace intervention. The fidelity score for both integrated care and the participatory workplace intervention was sufficient, although communication between members of the multidisciplinary team was limited. Participants were generally satisfied with the intervention. *Conclusions* This process evaluation shows that our intervention was not entirely implemented as intended. The integrated care was not delivered to enough participants, but for the intervention components that were delivered, the fidelity was good. Communication between members of the multidisciplinary team was limited. However, the participatory workplace intervention was implemented successfully, and participants indicated that they were satisfied with the intervention.

## Introduction

Rheumatoid arthritis (RA) is a chronic inflammatory disease severely impacting participation in daily activities such as work [1, 2]. Patients with RA are more prone to becoming permanently work disabled than the general population [3, 4]. Reduced at-work productivity and permanent work disability are common among patients with RA, leading to high costs [4–6]. Furthermore, participation in paid employment has an valuable meaning for patients with RA [7]. To support patients with RA to continue working and maintain and improve work functioning, the Care for Work intervention was developed. The Care for Work intervention consists of integrated care coordinated by a clinical occupational physician, and a participatory workplace intervention conducted by an occupational therapist.

With an evaluation of an intervention, effects of the intervention can be established. These effects are however dependent on the implementation of an intervention. Randomised controlled trials (RCT) are recognized as the golden standard for evaluating interventions [8]. The control group in an RCT makes it possible to distinguish between change over time and an actual effect of an intervention [8]. Alongside an RCT it is vital to investigate whether or not an intervention is carried out as intended, in order to place study findings into context [9]. The degree to which an intervention is performed as intended influences the extent to which the intervention has the opportunity to affect outcomes; very poor implementation of an intervention might lead to no effects on outcomes [10, 11]. Collecting data about the implementation of an intervention is furthermore important to prevent a Type III error. A Type III error might occur when researchers conclude that an intervention was not effective, while the lack of beneficial study findings was due to poor implementation of the intervention and not due to the working mechanism behind an intervention [12].

A process evaluation is a study in which the process of implementation of the intervention is investigated [13, 14]. A process evaluation can shed light on the success and failure of the application of an intervention, and on whether an intervention was delivered as planned [15–18]. Furthermore, the information obtained from a process evaluation can be used to improve the implementation of an intervention before implementing an intervention on a broader scale [19, 20]. However we should be cautious because participants in an RCT are not representative for the target group of an intervention on a large scale.

Our aim is to perform a process evaluation alongside an RCT of the Care for Work intervention to investigate whether the intervention was implemented as planned and whether patients were satisfied with the intervention, and hence study the feasibility of the intervention.

## Methods

This process evaluation was carried out alongside an RCT on the effectiveness of the Care for Work intervention program to maintain and improve work productivity for workers with RA [21]. RA patients were recruited from Reade (formerly the Jan van Breemen Institute), Amsterdam, the outposts of Reade, and the department of rheumatology of the VU University Medical Center, Amsterdam, the Netherlands. The medical ethics committees of the participating hospitals approved the study and all patients signed informed consent. More details about the design of the Care for Work study can be found elsewhere [21].

### Population

All patients that were randomized into the intervention group (n = 75) participated in the process evaluation. Inclusion criteria were: (1) diagnosis of RA; (2) aged between 18 and 64 years; (3) having a paid job (either paid-employment or self-employment); (4) working at least 8 h per week; and (5) experiencing difficulties in functioning at work. Patients could not participate in case of severe comorbidity, when they were unable to read or understand Dutch language, or when they had taken more than 3 months of sick leave at time of inclusion.

### Intervention

The intervention program consisted of two components which complemented each other; integrated care and a participatory workplace intervention. Both are described below.

#### Intervention Component 1: Integrated care

Integrated care was provided by a multidisciplinary team. This team consisted of a trained clinical occupational physician (who acted as care manager), a trained occupational therapist, and the patients’ own rheumatologist and occupational physician.

The care manager had an intermediate role between clinical and occupational care. He was responsible for the planning and coordination of care, and for communication between all members of the multidisciplinary team, the patient’s supervisor and general practitioner.

The patient visited the care manager within 1 week after randomisation. The care manager started with history taking and physical examination. History taking aimed to identify functional limitations at work and factors that could influence functioning at work. By the end of the first consultation, the care manager proposed a treatment plan, and sent the treatment plan to the other members of the multidisciplinary team. The patient visited the care manager again after 6 and 12 weeks to evaluate and if necessary adjust the treatment plan.

#### Intervention Component 2: Participatory Workplace Intervention

The workplace intervention concerned workplace adaptations and required active participation and strong commitment of both the patient and supervisor. The workplace intervention was based on methods used in participatory ergonomics [22–24]. The workplace intervention was coordinated by the trained occupational therapist, and executed by the patient and the patients’ supervisor. The aim of the workplace intervention was to achieve consensus between patient and supervisor concerning feasible solutions for the obstacles for functioning at work. After consensus regarding the solutions, the occupational therapist, patient, and supervisor agreed on an action plan to implement these solutions. Responsibility for implementing the plan of action was put on the patient and the patients’ supervisor’s account as much as possible. After four weeks, the occupational therapist evaluated whether the solutions had been implemented at the workplace.

### Data Collection

The data for this process evaluation were collected from medical records kept by the care manager and occupational therapist, and questionnaires completed by the patients before the start of the implementation and after 6-months of follow-up. In the medical records, care managers and occupational therapists kept notes of their contacts with the patient, the treatment plan as proposed by the care manager, and the action plan as created by the occupational therapist, the patient and the patients’ supervisor. Patients completed a questionnaire consisting of questions about whether the solutions proposed during the participatory workplace intervention were implemented. Furthermore, the questionnaire consisted of questions concerning their experiences with the care manager, occupational therapist, and their satisfaction with the intervention program. The care managers completed a questionnaire concerning the extent to which they communicated with the other members of the multidisciplinary team.

### Process Measures

#### Implementation of the Intervention Program

Implementation concerns the extent to which the intervention was delivered as planned. To describe the process of implementation, we used the concepts recruitment, reach, dose delivered, dose received, and fidelity of the framework proposed by Linnan and Steckler [25]. The process measures as used in this study are described in Table 1. Procedures used to recruit participants were described. Reach was addressed at participant level. Reach concerns the proportion of the intended target audience that participates in the intervention. As we performed a randomised controlled trial, 50 % of the participants in the trial were randomised into the intervention group. The number of patients invited to participate in the trial was registered, as well as the number of patients potentially interested. We furthermore listed the number of participants in the intervention group, and reasons for non-participation.

**Table 1:** Process measures

Concept	Definition	How was this measured?
Recruitment	Procedures used to recruit participants	Description
Reach	The proportion of the intended target audience that participates in the intervention	During the study, we registered the number of patients we invited, patients who eventually participated, and reasons for non-participation
Dose delivered	The amount of meetings planned according to the protocol by the intervention providers	We registered all planned meetings
Dose received	The extent to which participants actively engage with the intervention program	In the patient questionnaire, we asked participants whether they had implemented the solutions as proposed during the participatory workplace intervention
Fidelity	The extent to which the intervention was delivered as prescribed by the intervention protocol	Analysis of meeting notes as registered in medical records
Satisfaction	Satisfaction with the intervention program	We asked participants about their satisfaction by means of patient questionnaires

Dose delivered refers to the amount of meetings planned according to the protocol by the intervention providers. We registered whether the intake, 6- and 12-weeks evaluation by the care manager, the workplace intervention and evaluation by the occupational therapist took place. The intake was offered to all patients in the intervention group. Participants were only invited for the 6- and 12 weeks evaluation, and the workplace intervention if the intake took place. So, the dose delivered for these three intervention components was calculated by dividing for example the total number of 6-weeks evaluation meetings by the number of participants that took place in the intake. Participants were only invited for the evaluation by the occupational therapist if the workplace intervention took place. Dose delivered for the evaluation by the occupational therapist was therefore calculated by dividing the total number of evaluations by the occupational therapist, by the total number of workplace interventions offered. We furthermore registered whether the patients’ supervisor was present during the workplace intervention. Finally we calculated the mean dose delivered for the integrated care component and the participatory workplace intervention, by calculating the mean of all planned meetings per participant.

Dose received concerns the extent to which participants actively engage with the intervention program. We asked the participants whether they had implemented the solutions from the workplace intervention, and expressed this as a percentage (i.e. by dividing the number of implemented solutions by the total number of solutions that was agreed upon from the workplace intervention). All obstacles and solutions as proposed during the workplace intervention were classified based on the ergonomic abstracts classification scheme [26]. The classification categories were: performance-related factors; task-related factors; display and control design; workplace and equipment design; environment; and work design and organisation. Obstacles and solutions for functioning at work were classified by two researchers independently. Disagreements between the researchers were discussed to reach consensus. If there was no consensus, a third researcher was consulted to reach consensus.

Fidelity is a quality measure which refers to the extent to which the intervention was delivered as prescribed by the intervention protocol. For each participant, the meeting notes were registered in medical records. Two independent researchers recorded whether all components of the intervention were performed according to protocol. A list of intervention components was created in order to perform the scoring. This list consisted of all intervention components that were listed in the protocol. For example if limitations in functioning at work were discussed during the intake of the patient in the intervention by the care manager. Disagreements regarding the scoring between the researchers were discussed. If there was no consensus, a third researcher was consulted to reach consensus. A fidelity score was calculated separately for the integrated care component, and for the participatory workplace intervention. We calculated the fidelity score as a percentage. For example, we calculated the fidelity score for the participatory workplace intervention by dividing the number of intervention components that were delivered according to the protocol, by the total number of intervention components. When all quality measures of the intervention were performed according to protocol, a fidelity score of 100 % was reached.

Data concerning the extent to which the care managers communicated with other members of the multidisciplinary team were based on questionnaires completed by the care managers. The questionnaire contained items about all communication components of the protocol. For example, we asked the care managers if they had sent the treatment plan to the rheumatologist of the patient. These questions could be answered by four categories ranging from 1 to 4; never, sometimes, often or always (for every patient).

#### Satisfaction

Satisfaction with the intervention program was investigated by a questionnaire as part of the 6-month follow-up measurement. Whether employees were satisfied with their consultations with the care manager and occupational therapist was measured with two scales of the Patient Satisfaction with Occupational Health Services questionnaire (PSOHSQ); (1) being taken seriously as a patient during the last visit (6 items), and (2) trust and confidentiality during the last visit (3 items) [27]. Scores for the PSOHSQ are expressed as a score ranging from 0 to 4, a higher score indicates higher satisfaction. We furthermore asked the employees to give a score of one to ten to their contact with the care manager and occupational therapist, with ten indicating highly satisfied. We asked the patients to indicate whether they would recommend the intervention program to others (yes/no/maybe). We also asked patients about their satisfaction with the solutions discussed during the workplace intervention, with three items; whether they felt they had enough influence on the choice of the solution (yes/no), whether they were satisfied with the solutions (score 1–5, 1: not at all satisfied, 5: very satisfied), and which effect the solutions together had on their functioning (obstructed/no effect/promoted).

### Data Analysis

The data were analysed by means of descriptive statistics (mean, standard deviation, median, percentage). Excel 2010 and SPSS 20.0 (SPSS Inc., Chicago, IL, 2011) were used for the descriptive and statistical analyses.

## Results

### Recruitment and Reach

Eligible patients received an information letter about the project from their own rheumatologist. This letter included a reply card which patients could send to the research team by mail to indicate whether they were interested in participating in the study. Interested patients were contacted by the researcher by telephone. Additional information about the study was given, and the eligibility of the patient was checked. If a patient was willing to participate and met all selection criteria, the researcher planned a face-to-face appointment with the patient. During this appointment, the patient signed informed consent, and completed the baseline questionnaire. Next, randomisation was performed.

**Fig. 1:**
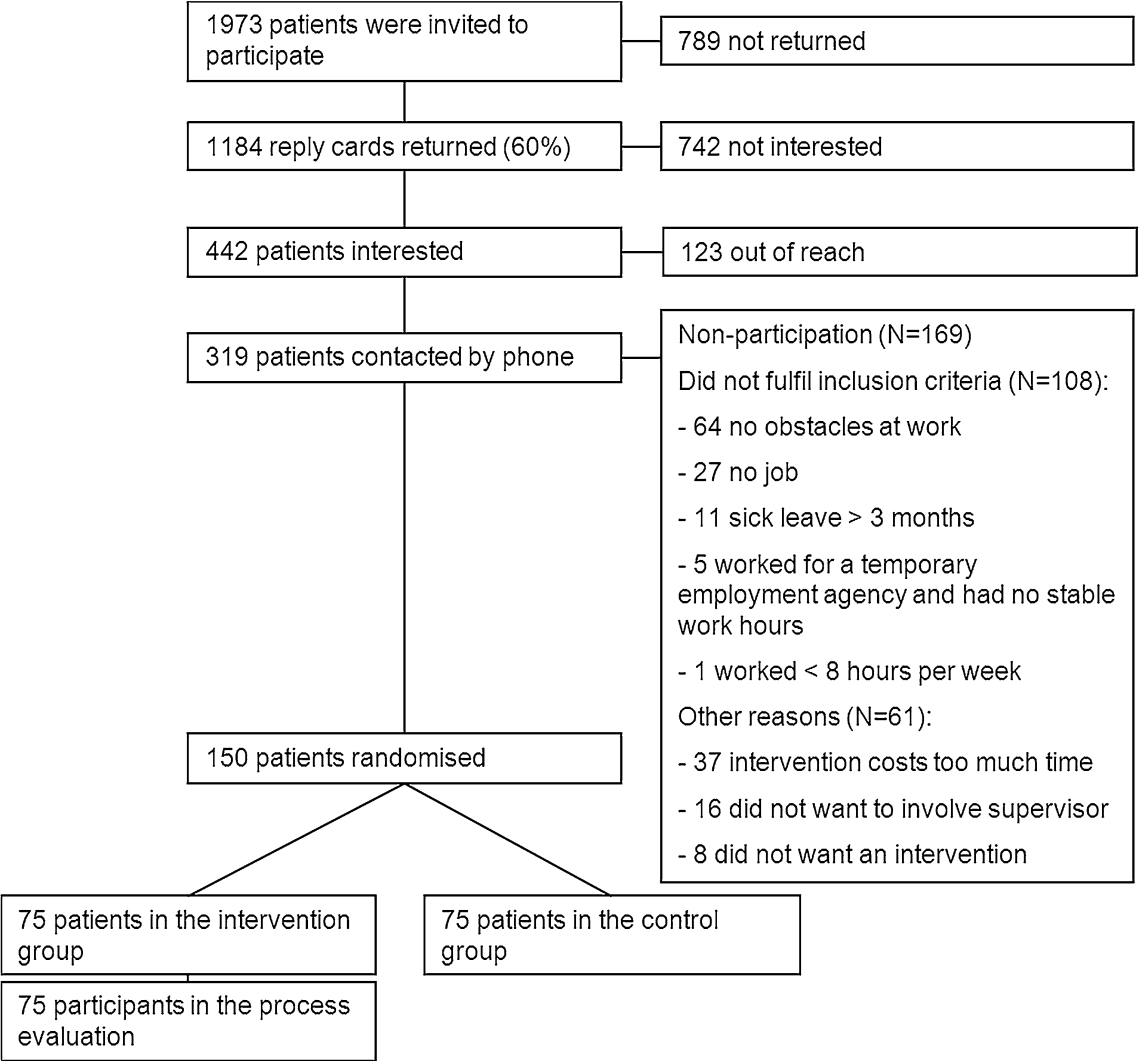
Flow diagram

Figure 1 shows the flow diagram of RA patients in the Care for Work study. We invited 1973 patients to participate. We received 1184 reply cards from patients, of which 424 patients were potentially interested. We contacted 319 patients by phone, 169 of these could not participate or were unwilling to participate. The main reason for non-participation was no perceived obstacles at work, followed by time restrictions related to the intervention. In fact, 108 patients could not participate based on the in- and exclusion criteria (63.9 %), and 61 patients refused to participate (36.1 %). Finally, 150 patients were randomised, of which 75 were randomised into the intervention group. Characteristics of the 75 patients in the intervention group are described in Table 2.

**Table 2:** Characteristics of participants in the intervention group N = 75

Variable	Intervention n = 75
Gender^a^	
Male	12 (16 %)
Female	63 (84 %)
Age^b^	
Years	49.8 (8.6)
Education^a^	
Low	16 (21 %)
Middle	22 (29 %)
High	37 (49 %)
Comorbidity present^a^	
No	29 (39 %)
Yes	46 (61 %)
Duration since diagnosis^b^	
Years	10.9 (9.1)
Job satisfaction^a^	
Satisfied	46 (61 %)
Not satisfied	29 (39 %)
Job contract or self-employed^a^	
Job contract	61 (81 %)
Self-employed	14 (19 %)

^a^n (%)

^b^m (sd)

### Dose Delivered

Table 3 shows dose delivered of the intervention. Of all participants randomised into the intervention group, 81.3 % took part in the intake. Of the participants who took part in the intake, 37.7 % took part in the 6-weeks evaluation, and 13.1 % took part in the 12-weeks evaluation by the care manager. Of the participants who took part in the intake, 85.3 % took part in the workplace visit by the occupational therapist. The evaluation of the occupational therapist was offered to 67.3 % of participants who took part in the workplace visit. When a workplace visit took place, the supervisor was present at 88.5 % of the workplace visits. Finally, the mean dose delivered for integrated care was 46.7 % and for the participatory workplace intervention 80.6 %.

**Table 3:** Dose delivered and dose received (classification of obstacles and solutions)

	N	Score (%)
*Dose delivered*		
Integrated care		
First consultation	61/75	81.3
6-weeks evaluation	23/61	37.7
12-weeks evaluation	8/61	13.1
Total score dose delivered integrated care		46.7
Participatory workplace intervention		
Workplace visit	52/61	85.3
Evaluation	35/52	67.3
Supervisor present at workplace visit?	54/61	88.5
Total score dose delivered workplace intervention		80.6

Category	Obstacles n (%)	Solutions n (%)
*Dose received*		
Performance related factors	45 (34 %)	19 (14 %)
Task related factors	24 (19 %)	5 (4 %)
Display and control design	15 (12 %)	13 (10 %)
Workplace and equipment design	33 (25 %)	41 (30 %)
Environment	1 (1 %)	0 (0 %)
Work design and organisation	11 (9 %)	59 (43 %)
Economic impact of the system	1 (1 %)	0 (0 %)
Other	0 (0 %)	0 (0 %)

### Dose Received

Patients reported that 69.5 % of all solutions proposed during the workplace visit had been implemented at the workplace. Table 3 shows that most obstacles were performance related, such as fatigue and a lower energy level, followed by obstacles with workplace and equipment design, for example sitting. Most solutions to the obstacles are classified to work design and organisation, for example a day planning with more breaks, or to discuss work tasks and planning with the supervisor more frequently. This was followed by solutions related to workplace and equipment design, such as adaptations to an office chair and desk.

### Fidelity

The fidelity score, which refers to the extent to which the intervention was delivered as planned by the intervention protocol, was 85.7 % for integrated care (Table 4). The fidelity score was a bit lower for the participatory workplace intervention, specifically 68.7 %. Based on the questionnaire filled out by the care managers, we found that 45.8 % of the communication to members of the multidisciplinary team was delivered as planned. In most cases, the rheumatologist was informed about the treatment plan of the care manager, but the care manager failed to inform the rheumatologist about the workplace intervention. Communication with the patients’ own occupational physician was also poor, while the general practitioner of the patient was never informed by the care manager.

**Table 4:** Fidelity

Intervention component	Fidelity score (%)
Integrated care	85.7
Participatory workplace intervention	68.7
Communication care manager with others	
Send treatment plan to rheumatologist	83.5
Send treatment plan to occupational physician	50.0
Inform occupational physician by phone	16.5
Send treatment plan to occupational therapist	100
Inform participant’s supervisor	50.0
Inform rheumatologist about workplace visit	16.5
Inform occupational physician about workplace visit	50.0
Inform general practitioner about treatment plan and workplace visit	0

### Satisfaction

Patients scored 3.1 (SD 0.7) out of a possible 4 on the scale ‘being taken seriously’ of the PSOHSQ for the care manager, and 3.0 (SD 0.6) for the occupational therapist. Concerning trust and confidentiality, patients scored 2.4 (SD 1.5) for the care manager. Patients gave the care manager a mark of 8.2 (SD 1.3), and a 7.9 (SD 1.3) for the occupational therapist. Most patients (67.1 %) would recommend the intervention program to others, while 11.0 % would not. The remaining patients (21.9 %) might recommend the intervention program.

In 87.2 % of cases, patients felt they had had sufficient influence on the choice of the solutions during the workplace intervention. On a scale of 1–5 (not satisfied to very satisfied), they were satisfied with the solutions with a score of 3.5 (SD 1.1). Patients indicated that the solutions promoted their functioning (66.0 %), that the solutions had no effect on their functioning (29.8 %), or that the solutions obstructed their functioning (4.3 %).

## Discussion

### Main Findings

Our objective was to perform a process evaluation of the Care for Work intervention to assess whether the intervention was implemented as planned. We furthermore investigated the satisfaction of the patients with the intervention program. Overall, the implementation of the participatory workplace intervention was adequate. The implementation of integrated care was less successful. We will compare our findings with findings from other studies, although in the field of rheumatology, there are to our knowledge no process evaluations available on comparable interventions.

We were not able to determine the actual reach of our intervention. The rheumatologists did not have information about the work status of invited patients, as a consequence, the letter was also sent to patients within the specified age group, but without a paid job. This might explain the high number of patients who did not send back the reply care or send back the reply card indicating that they were not willing to participate (40 and 38 % out of all invited patients, respectively). Because we do not know the percentage of patients in our invited sample who had a paid job, we do not know how many of the invited patients actually belong to the intended target audience of our intervention, and we cannot calculate the actual reach. Work disability rates among patients with RA differ tremendously between studies [28, 29], we were therefore also not able to make an estimation of the number of patients with a paid job in our invited sample. About half of the patients we were able to contact by phone, eventually did not participate. This percentage is comparable to another study in which a similar intervention was offered to workers with low back pain [30].

The intervention was delivered to a lesser extent than was intended. Only 81.3 % of patients took part in the initial intake and started the intervention, compared to 92.5 % in a comparable study [30]. The other intervention components could only take place when the intake was carried out, so because almost 20 % of patients did not participate in the intake, they did not start the intervention. The evaluations by the care manager were delivered in a few cases only. The workplace intervention was delivered to 85.3 % of patients who had started the intervention, and the evaluation was offered to more than half of the participants. In most cases, the supervisor was present during the workplace visit, which was a very important part of our intervention. In another intervention study which also consisted of, amongst others, a meeting between the worker and supervisor, it was found that this meeting only took place for 10 % of the participants [31]. Eventually, the mean dose delivered for integrated care was 46.7 %, and for the workplace intervention 80.6 %. The low percentage for dose delivered was mostly due to the low delivery of the intake and the evaluations by the care manager.

We found that 69.5 % of the solutions proposed during the workplace intervention were implemented. This percentage is comparable to another study in which the participatory workplace intervention was evaluated (72 %) [30]. In the comparable study, the intervention was offered to workers on sick leave. We therefore suspected the percentage of implemented solutions to be lower in our study, since one could argue that the need to implement solutions is higher and more urgent when a worker is on sick leave. Our study results show however, that the percentage of implemented solutions is comparable in a study sample of workers who are not on sick leave. It has been proposed before that the implementation of solutions might also be related to whether the workers suffers from a chronic disorder or not. The Lambeek study on chronic low back pain had an implementation rate for the solutions of 72 % [30], while two other studies on (sub) acute low back pain had implementation rates of the solutions of only 50 % [22, 32].

The fidelity score for integrated care was high, which means that of the intervention components that were delivered, the quality was good. The fidelity score for the participatory workplace intervention was a bit lower, but still 68.7 %, which is satisfactory. The communication of the care manager with other caregivers involved was executed poorly. Previous research has also emphasized that communication between an occupational physician and other care givers is poor [33]. Given that this RCT was carried out in a controlled environment, the communication efforts executed are very poor. Thereby, the integration of the care offered to our participants failed, and hence the linkage of all care givers towards one treatment goal was not achieved. It has been described by previous studies that it is difficult to enhance interprofessional collaboration [34, 35].

Despite the previously described shortcomings in the implementation of the intervention, patients were satisfied with the intervention. They felt taken seriously by the care manager and occupational therapist, and they rated them with high marks. The issue of trust in an occupational physician has been documented before [36]. Although the care manager who delivered the intervention in our study was not linked to the employers of our patients, and hence was independent, our patients still had concerns about trust and confidentiality of the care manager.

### Strengths and Limitations

This process evaluation provides insight into the implementation of an intervention program, consisting of integrated care and a participatory workplace intervention. We collected data for this process evaluation from both the patients, as well as the intervention providers. The components of this process evaluation were collected by means of self-reported data; patients filled out a questionnaire, and intervention providers wrote reports, and hence no objective data was collected. Furthermore, in this process evaluation, only quantitative data was collected. Qualitative data could add a more context-specific insight into the implementation, which would help to interpret our findings [37].

Because we were not able to calculate the actual reach of our intervention, we cannot fully determine the representativeness of our study sample. In RCT studies, the study sample is generally not representative of the target group which might lead to bias, since typically, motivated patients participate in research projects. In our sample, especially men might be underrepresented. We based our fidelity scoring on medical records kept by the care manager and occupational therapist; we were not there during consultations. Therefore, we cannot rule out bias. Furthermore, we asked the care manager about their communication efforts by sending them a questionnaire. This might have led to socially desirable answers, since they were aware what their efforts should have been according to the protocol. Furthermore, there was one care manager who performed most intakes and evaluations. Therefore, a lot of the results depended on the skills of this specific care manager. The possibility or recall bias is negligible in our study design, since we asked participants about their experiences with the intervention shortly after the intervention.

We have not collected data about the reasons why our intervention, and especially integrated care, was not delivered as planned in the study protocol. In future research it is very important to study these issues, to overcome them. We found that communication between members of the multidisciplinary team was limited. To implement an intervention consisting of integrated care, it is important to find out why there was only little communication. If there are practical reasons for this, these barriers have to be addressed in order to improve dose delivered by the intervention providers.

### Implications

This study shows that a process evaluation can provide essential information about the implementation of an intervention, which is vital if an intervention is to be implemented in practice. We made use of quantitative data. For future process evaluations, we recommend to use both quantitative and qualitative data. Qualitative data can add insight information and lead to an explanation of findings. Given that our intervention was not delivered to the extent we aimed for, we recommend to look critically to the intervention protocol with the intervention providers. Our intervention was not delivered to the extent we aimed for. Especially for the integrated care component, a large number of participants have not received the evaluations. It is important to discuss with the intervention providers why the evaluations were delivered only seldom, and why the intake was delivered to only 81.3 % of the participants. Whether this occurred by for example administrative issues, it should be addressed, and consequent adaptations to the protocol are needed. Furthermore, we were not able to integrate care towards our patients. There was only little communication between members of the multidisciplinary team. Previous research has also shown that communication between medical specialists is difficult [33]. For medical specialists working under pressure, communication with other medical specialists might be very difficult to establish. If an intervention is to be implemented aiming to integrate care, opportunities for communication should be embedded in the daily practice of medical specialists involved, such as specific planned time points for conference calls.

## Conclusions

This process evaluation shows that our intervention was not entirely implemented as intended. The integrated care was not delivered to enough participants, but for the intervention components that were delivered, the fidelity was good. Sufficient communication between members of the multidisciplinary team could not be established by the care managers. However, the participatory workplace intervention was implemented more successfully. The workplace intervention was delivered satisfactorily, and participants indicated that they implemented the solutions from the action plan to an adequate extent. Participants indicated that they were satisfied with the intervention, and that they would recommend the intervention to others.
